# Autonomous Sensor System for Low-Capacity Wind Turbine Blade Vibration Measurement

**DOI:** 10.3390/s24061733

**Published:** 2024-03-07

**Authors:** Diego Muxica, Sebastian Rivera, Marcos E. Orchard, Constanza Ahumada, Francisco Jaramillo, Felipe Bravo, José M. Gutiérrez, Rodrigo Astroza

**Affiliations:** 1 Facultad de Ingeniería y Ciencias Aplicadas, Universidad de los Andes, Santiago 7620001, Chile; dmuxica@miuandes.cl (D.M.); fbravo1@miuandes.cl (F.B.); jmgutierrez@miuandes.cl (J.M.G.); 2 DCE&S Group, Department of Electrical Sustainable Energy, Delft University of Technology, 2628 CD Delft, The Netherlands; 3 Department of Electrical Engineering, Centro de Energía, Universidad Católica de la Santísima Concepción, Concepción 4090541, Chile; 4 Department of Electrical Engineering, Faculty of Physical and Mathematical Sciences, University of Chile, Av. Tupper 2007, Santiago 8370451, Chile; morchard@u.uchile.cl (M.E.O.); coahumad@uchile.cl (C.A.); francisco.jaramillo@ing.uchile.cl (F.J.)

**Keywords:** accelerometer-based sensor networks, condition monitoring, data acquisition, modal analysis, structural health monitoring, wind turbines

## Abstract

This paper presents the design, implementation, and validation of an on-blade sensor system for remote vibration measurement for low-capacity wind turbines. The autonomous sensor system was deployed on three wind turbines, with one of them operating in harsh weather conditions in the far south of Chile. The system recorded the acceleration response of the blades in the flapwise and edgewise directions, data that could be used for extracting the dynamic characteristics of the blades, information useful for damage diagnosis and prognosis. The proposed sensor system demonstrated reliable data acquisition and transmission from wind turbines in remote locations, proving the ability to create a fully autonomous system capable of recording data for monitoring and evaluating the state of health of wind turbine blades for extended periods without human intervention. The data collected by the sensor system presented in this study can serve as a foundation for developing vibration-based strategies for real-time structural health monitoring.

## 1. Introduction

The search for clean and sustainable alternatives to meet the ever-increasing energy needs of our society led to remarkable advancements and developments in renewable energy technologies. Among the different alternatives, wind energy has become one of the main technologies to compete with conventional energy sources, making this form of renewable energy a significant component of current and future energy supply systems. Given the sustained technological growth, the cumulative installed capacity increased exponentially to reach about 850 GW in 2021 [1], and nowadays, large wind turbines (WTs) (with capacities of up to 8–16 MW) are widely being installed in power distribution networks [2,3].

For instance, in 2021, wind energy contributed to approximately 7% of the total electricity generation [1]. Hence, it is becoming a significant player in the operation of the modern grid system. Therefore, every year is becoming more critical to guaranteeing efficient, reliable, and safe wind power generation. Reliability is an important index as it could lead to a further reduction in the overall costs of renewable energy resources, given the long-term investment they imply and their expensive reparation costs [3,4]. Hence, the structural integrity of WTs should be guaranteed [5].

Nevertheless, maintenance of WTs has become a challenging endeavor. Considering their trend toward greater sizes, their complexity (which encompasses electric, electronic, mechanical, and structural components) and the fact that they are slender civil structures typically operating in harsh environments (e.g., isolated mountainous or turbulent sea regions) lead to massive structures that are exposed to large dynamic loads and enormous mechanical tensions and strains. These issues have sparked interest in the prognosis and diagnosis of structural damage to ensure the safe and reliable operation of these systems [6,7,8,9].

Numerous studies for preventing unscheduled downtime have suggested monitoring WT blades, and different techniques have been proposed [10,11]. Nowadays, different well-established methods can be found in the industry for monitoring the structural health of the blades, which are based on the usage of strain sensors, deflection sensors, digital image correlation, accelerometers, accousting emission, and guided waves, showing different efficacy and capability regarding damage detection in large-scale wind turbine blades. Strain and acoustic emission methods are more prone to detecting local damage, while vibration-based methods can be more effective for global damage detection [12]. Additionally, regarding signal processing studies, methods utilizing piezoelectric impact sensors and acoustic emission sensors have been implemented for the fault diagnosis of WTs [13]. For instance, in [14], blade failure was detected through ultrasonic testing. Other works proposed using optical method sensors to detect abnormal states in a rotor system as a whole through fiber-optic sensing [15], employ cost-effective three-dimensional digital image correlation to monitor the blades’ structural health [16], or use a laser-Doppler vibrometer system for modal parameter identification [17]. Finally, more recent works have proposed using a Doppler radar sensor for feature extraction and fault detection [18] or blade deflection using novel wireless inertial measurement units [19].

According to the authors of [20], integrating a structural health monitoring system in a WT by using vibration sensors to monitor the blades’ integrity shows great promise. However, such systems remain costly and power-intensive due to challenges in data collection, processing, storage, and transmission [21]. Moreover, the remote locations of wind turbines demand reliable and robust operation with minimal maintenance. To ensure stable operation, systems designed for smaller turbines must incorporate an autonomous power source and minimize power consumption to support a robust sensing mechanism.

This paper proposes a cost-effective and reliable sensor architecture based on microelectromechanical system (MEMS) accelerometers attached to the blades’ surfaces. The system is capable of monitoring the condition of WT blades for extended periods of time without human intervention. The developed system was installed in two operational 5 kW turbines, one of which was located in Chile’s Aysen region, where the colder oceanic climate provides low temperatures, abundant precipitation, and ideal conditions for wind energy generation (i.e., strong and constant wind speeds). As the system needs to rotate with the blades and avoid error sources due to a slip ring connection, the system is powered by a small battery pack that is recharged using monocrystalline photovoltaic (PV) modules. This, combined with the low light during the austral winter, creates challenging conditions. Despite these challenges, the system is able to perform and transmit near real-time measurements and provide insights into the blades’ modal vibration characteristics throughout the year.

The remainder of this paper is organized as follows. A general overview of the monitoring system, describing the microcontroller employed, the sensors involved, specific components that complete the system, and the energy source, are described in Section 2. The system’s operation as well as the design considerations made during installation are explained in Section 3. Experiments conducted to evaluate the robustness and accuracy of the sensor system and the outcomes of the installations carried out in Chile are discussed in Section 4. Finally, the conclusions are presented in Section 5.

## 2. Monitoring System Overview

**Figure 1:**
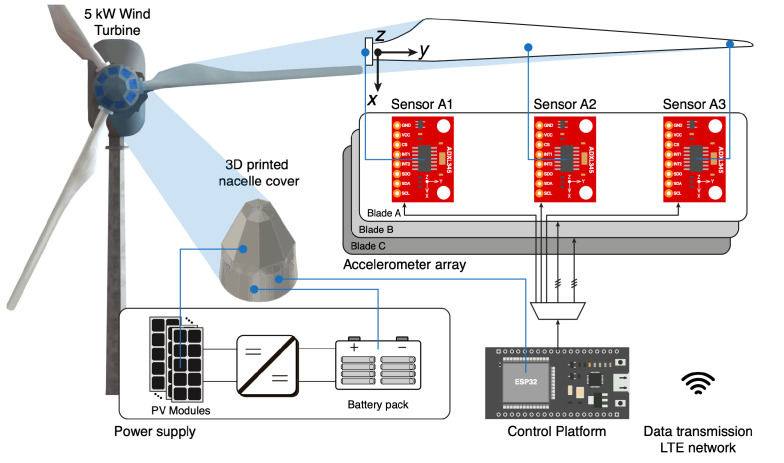
Hardware disposition overview of the embedded damage detection autonomous system for WT blades.

The proposed autonomous system was developed to address the problem of monitoring the state of health of WT blades. During assembly, sensors were deployed on a 5 kW variable pitch WT (WT1) installed in the Alto Baguales wind park in Chile’s Aysen region, while a twin system was installed on an already operational WT (WT2) at Universidad de los Andes campus in Santiago, Chile. This led to the development of a modular system capable of easily changing components such as the microcontroller unit or modifying the sensing network. A high-level overview of the proposed architecture is presented in Figure 1, where a network of on-blade three-axis accelerometers is installed to capture the vibrations of the system. The array is connected to a microcontroller, which centralizes the operation of the sensing network. The collected data are transmitted for post-processing through the mobile network, aside from being stored locally as a backup. The proposed monitoring system is powered by an array of PV panels and a Li-ion battery pack to ensure its autonomous remote operation. It is noted that in this proof-of-concept stage of the instrumentation system, accelerometers and cables are installed on the outer surface of the blade. However, the idea in a final commercial solution is to install the components on the interior surface of the blade.

Similar structural health monitoring systems typically measure vibrations in a 0–20 Hz bandwidth, while the principal modes of the structure are in the range of 0–10 Hz [22]. It is noted that the principal modes of the blades being analyzed in this case were in the range of 0–80 Hz [23]. Therefore, vibrations would be measured with a sampling frequency of 200 Hz to capture all the essential information for post-processing. It is noted that in this study, the raw acceleration data recorded by the instrumentation system were pre-processed by applying a fourth-order Butterworth filter with cut-off frequencies from 0.1 to 90 Hz. This choice guaranteed no aliasing for the acceleration signals and that the most important vibration modes of the blades could be properly identified. The previous studies determined the mode shape that helped define the sensor positions along the blade in the flapwise and edgewise directions.

The WT1 location is crucial to validating the system in a relevant environment, which presents challenging weather conditions throughout the year, particularly frequent rain (657.2 mm yearly average), snow throughout the winter, wide temperature variation (ranging from −8 °C to 28 °C), and a narrow clear sky window during winter, being below 25% on average. Considering these aspects, the following components were employed.

### 2.1. Microcontroller

The monitoring system employs a board based on the ESP32. The Espressif Systems ESP32 is a low-cost, low-power microcontroller with integrated Wi-Fi and Bluetooth capabilities. It has a dual-core processor, allowing it to handle multiple tasks simultaneously, and an internal flash memory, making it suitable for cache data storage. The ESP32 is commonly used in Internet of Things applications, such as home automation, sensor networks, and connected devices because it has multiple GPIO pins that can host up to three SPI channels. Given that the ESP32 has two cores, one core is dedicated to sampling the data from the nine sensors accurately with a sampling rate of 5 ms, while the other core is responsible for storing the data gathered in a 10 min window. A 10 min window was used since it could be sent comfortably through the cellular network in a short time. After capture, the system suspends measurements for 10 min, allowing the ESP32 to communicate the collected data files and any other files that were unable to be transmitted due to power or connectivity limitations.

Since the system is intended to function in harsh conditions, where the unpredictability of the weather will play a part in its operation, the ESP32’s software is designed to be reliable to ensure low maintenance and minimal downtime. Thus, the battery voltage is measured for estimating its state of charge, and the information transmission is delayed if it falls below a predetermined threshold. If the battery reaches a critical level, then all measurements are halted, allowing the system to recharge and continue operating when conditions improve.

**Figure 2:**
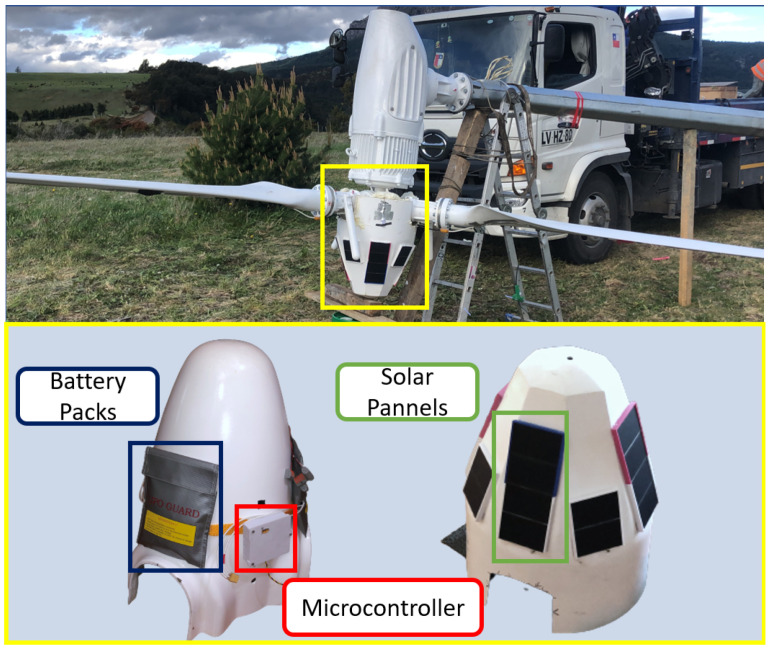
Overview of the nose hub and its components. They are distributed in such way that the hub maintains its balance while rotating. Most components are secured with bolts and a silicone-based glue to absorb vibrations.

To produce the necessary area for the sensors while avoiding changes to the WT, an additional fiberglass nacelle was fabricated. This structure, illustrated in Figure 2, would serve as the support for a series of solar panels that power the monitoring system. It also leaves a space between itself and the turbine which is big enough to accommodate an array of Li-ion batteries capable of storing extra solar energy and allows the system to operate continuously. To house the microcontroller, which was inserted into this same space, a polyethylene terephthalat glycol (PETG) container was printed.

### 2.2. Sensors

The monitoring system utilizes ADXL345 sensors (Analog Devices, Wilmington, MA, USA), compact triaxial digital accelerometers with low power consumption, a high resolution (13 bits), and a customizable amplitude range of ±16 g [24]. These accelerometers, characterized by a sensitivity of 141 LSB/g, provide precise measurements, while their stability is underscored by a low noise level of 0.25 LSB*_rms_*. Operating seamlessly across a broad temperature range from −40 °C to +85 °C, the ADXL345 maintains accuracy in diverse environmental conditions. This capacitive accelerometer, with a passband from 0.05 Hz to 1600 Hz, is capable of monitoring dynamic accelerations, low-frequency vibrations, static gravity accelerations, motion, and angles of inclination. Aside from that, the sensor has minimal power requirements, making it ideal for standalone operation by offering a 400 Hz sampling rate in low power mode.

As described earlier in Figure 1, each of the turbine’s blades is equipped with a set of three accelerometers. Each sensor requires five shared connections, since their measurements are transmitted through SPI, and a dedicated channel for chip selection, leading to a total of eight wires per blade. Consequently, a Cat7 LAN cable was utilized to connect the array of sensors. It is worth noting that future applications might consider having the accelerometers within the blade structure to minimize the aerodynamic disturbances caused by their size.

The peripheral components, including the sensors and SD card, were connected using the SPI protocol. This interface facilitates full-duplex communication between SPI devices following a master-slave architecture. In this set-up, four logic signals are utilized. By employing an individual chip select line, multiple slave devices can be selected, while the remaining three signals can be shared among independent slave configurations.

As previously mentioned, the primary objective was to distribute the tasks among the cores of the microcontroller. One core was dedicated to controlling the accelerometers, while the other core handled the logging of information obtained from the accelerometers onto the SD card. Since these tasks needed to be performed simultaneously, it was crucial to allocate each task to a separate SPI channel. This segregation was necessary to prevent interference or delays arising from variable writing times on the SD card.

Subsequently, a DHT22 temperature sensor was incorporated into the experimental set-up. This addition was motivated by the findings from previous modal identification studies, where it was observed that environmental conditions, especially the temperature, can introduce fluctuations in the estimated dynamic parameters [25,26].

### 2.3. Energy Harvesting and Storage

The optimization of energy consumption is one of the main challenges when designing a WT health monitoring system. The operational conditions of the system implies the use of an independent source, given the rotating nature of the elements. Thus, enabling a reliable connection from the pole to the center would require multiple mechanical components that could compromise system reliability [27]. Regarding independent sources, these can be separated between high variable output and low variable output.

Low variable outputs are able to provide highly predictable power to the system. In this category, piezoelectric devices, electromagnetic harvesters, and radio frequency harvesters can be distinguished. As with any other energy source, however, they have disadvantages. Piezoelectric devices are typically expensive, and because their output is alternating current, they must be rectified prior to use. Electromagnetic harvesters are cumbersome and comprise numerous movable parts prone to fatigue and failure. Finally, radio frequency energy requires a large antenna to capture small amounts of power [28], and since WTs are located far from densely populated areas, there will be little radio frequency energy available for harvesting.

Sources with low variable outputs are inappropriate for the designed monitoring system, unlike sources with variable outputs, such as PV or thermal energy harvesters. Thermal energy harvesters require constant variation to produce the required energy, making the power production more difficult to predict. Therefore, solar PV panels were selected as the power source. To ensure constant operation, a suitable battery pack was considered and sized to provide a steady supply to the system. A 39,600 mAH battery pack consisting of 18 Li-ion 18,650 cells was employed, enabling the system to operate uninterrupted for 3 days under no sunlight conditions.

## 3. System Deployment

### 3.1. Prototype Energy Assessment

The idea behind the proposed sensor system is to ensure a reliable health monitoring system with minimal requirements for both upkeep and maintenance. Previous works, like [29], showed that wireless connectivity is one of the most relevant sources of consumption. To guarantee its transmission capabilities, initially, a sensor node complemented by a base station designed similar to the one proposed by Polonello et al. [30] was tested. In this set-up, the sensor node at the hub would transmit data in small packages to a secondary microcontroller located in the turbine’s tower immediately after capturing it.

By placing the base station in the turbine’s tower, it enabled power from the utility grid, thereby confining the energy consumption challenge to the sensor node while ensuring a stable energy source for data transmission. Moreover, transmitting the data immediately upon measurement simplified the process in the microcontroller, freeing up resources for timekeeping. Additionally, removing the onboard microSD created more available pins, eliminating the need for a multiplexer for sensor selection.

As mentioned earlier, the prototype retrieves acceleration readings from nine sensors along the *x* and *z* axes (i.e., edgewise and flapwise, respectively) at a rate of 200 Hz for a duration of 10 min. Subsequently, the system enters a 10 min sleep mode frame to facilitate battery charging in low-light conditions. This cycle is repeated periodically unless the system enters the critical battery level zone, where more stringent power consumption protocols are implemented to prevent disconnection of the main board. To minimize power consumption, the system enters deep sleep immediately after completing the measurements. Deep sleep mode deactivates the CPUs, most of the RAM, and all clocked digital peripherals. However, some remain energized as the wake up source uses the internal clock to calculate the amount of time until the next measuring period. The system is reset before each cycle.

**Figure 3:**
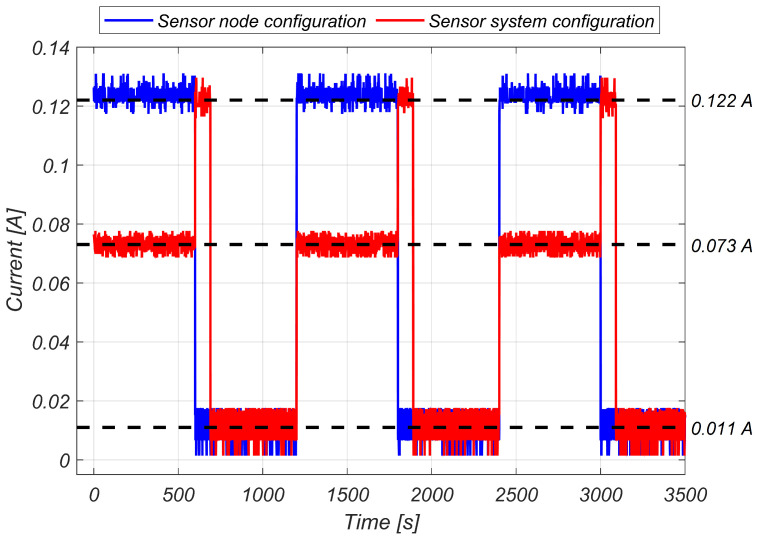
The graph shows the charge flow for both configurations. The higher consumption was due to the Wi-Fi module being used for data transmission. This led to an increase of 67.1% compared with normal operation using a base station. The lower consumption band shows the system in sleep mode, reducing consumption by 84.9%.

Comparing both architectures (refer to Figure 3), it is evident that a significant portion of power is consumed during data transmission. Additionally, transmitting the data in small packages at that frequency resulted in no significant difference in the maximum power utilized when sending the data to the main station compared with sending it directly from the node. This highlights the importance of minimizing the transmission time. Consequently, the most viable solution was to efficiently measure the data before transmission. To address this, the measurements were saved as binary data in text files, leading to a reduction in the total number of transmitted bits and subsequent power savings.

### 3.2. System Design and Installation

WT1 was installed in the southern region of Chile to assess the monitoring system’s performance in challenging weather conditions. Both WT1 and WT2 were identical three-bladed WT, featuring a rotor diameter of approximately 7 m and a hub height of 12 m.

Because the system would need to be installed in both a partially assembled (WT1) and fully assembled turbine (WT2), components were bundled into submodules to facilitate installation. Sensor strips were built with LLDPE-coated outdoor Cat7 LAN cables. The sensors were soldered together and encased in resin with 3D-printed molds to protect them from UV light and provide a more streamlined design. Each strip had three sensors one meter apart from each other. Subsequently, a more flexible LAN cable was used, which was capped with an RJ45 connector for ease of installation.

Beginning at the hub, sensors were affixed to the blades 80 cm apart, using hot-melt adhesive to transmit vibrations with minimal damping, and covered with a silicone-based sealant to prevent the sensors from coming loose during operation, aside from protection from the elements. The LAN cable was routed and secured using speed tape, which is resistant to water, solvents, and flames for brief periods and is capable of reflecting UV light and heat.

Under the secondary nose hub, where the controller was located, LAN cables were then routed. To connect the batteries, packs of six Li-ion batteries were placed in battery charging bags to protect them from the elements. Then, each bag was attached to a tie-down strap that was bolted to the primary hub. PV panel arrays were attached to the outer nose hub using a 3D-printed frame, bolts, and silicone sealant to prevent vibrations. These panels were connected to charge regulators in order to supply energy to the batteries.

## 4. Experimental Results

### 4.1. Sampling Rate Examination

To ensure accurate analysis of WT blades and potential prediction of premature damage, it is crucial to measure vibrations at a constant frequency. This not only enables reliable data acquisition but also facilitates effective comparison and analysis of the obtained measurements.

**Figure 4:**
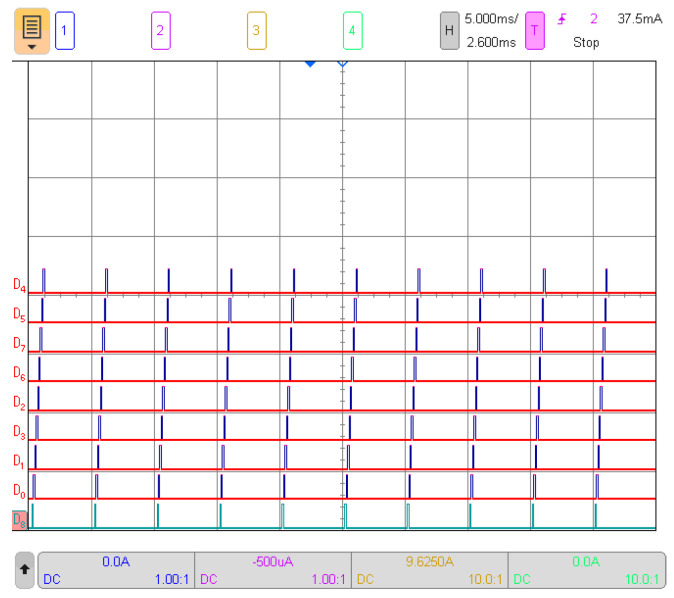
Oscilloscope capture illustrating sequential activation of nine digital channels with 5 ms division intervals.

Figure 4 demonstrates the synchronized engagement of each sensor at a precise 5 ms interval. The oscilloscope image illustrates the sequential activation of the nine sensors due to the usage of a common chip select channel in the SPI protocol, preventing simultaneous activation. Despite not being activated simultaneously, equal separation between measurements was maintained, enabling comprehensive data collection. The figure also reveals that sufficient time remained between sensor selections, allowing for potential inclusion of additional sensors in future implementations.

**Figure 5:**
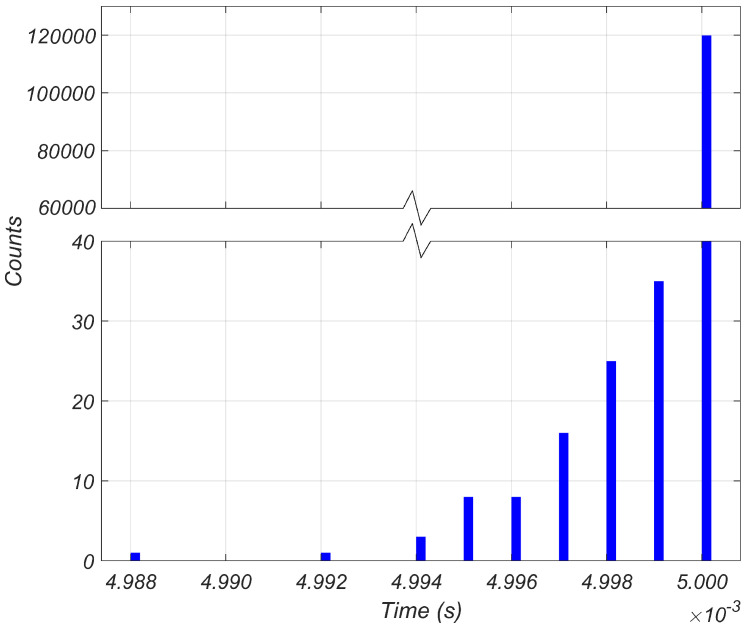
Histogram illustrating the timing of the conducted measurements.

To validate the 200 Hz sampling frequency, Figure 5 presents a histogram accompanied by a box plot visually depicting the precise timing of each measurement. The histogram provides a distribution of the measurement timings, with deviations not exceeding 0.24%. Additionally, the box plot offers key statistical insights, revealing that 99.9% of the measurements were captured with a 5000 μs spacing, while a small fraction of the values was captured at slightly higher frequencies. This visual representation confirms that the majority of the measurements were acquired at a constant frequency of 200 Hz. The consistent time interval maintained throughout the measurements ensures uniformity and accuracy of the acquired vibration data.

The ability to consistently measure at a constant frequency significantly enhances the reliability and integrity of the acquired vibration data. This precision empowers detailed analysis and accurate identification of any anomalies or patterns that may indicate premature damage in WT blades.

### 4.2. Monitoring Energy Storage

**Figure 6:**
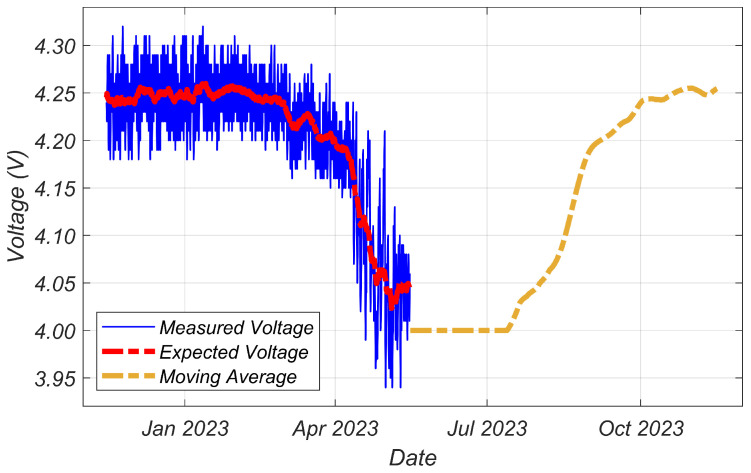
Battery voltage over time. The graph displays measurements taken up until the 15th of May. In addition to the raw measurements, the graph also features a moving average and the expected values for the remaining period of the year.

**Figure 7:**
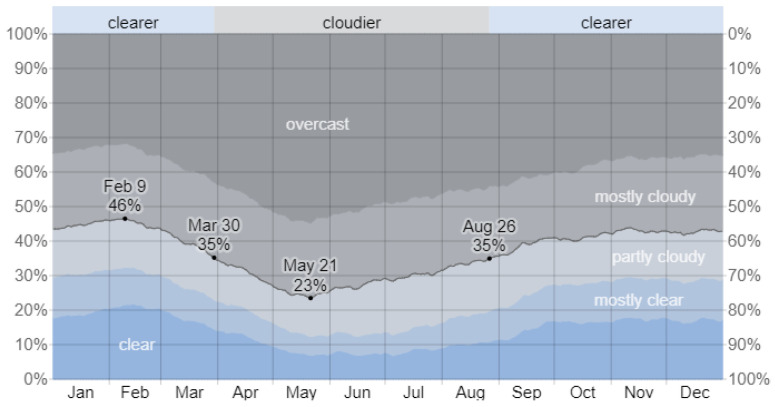
Cloud cover categories in Aysen throughout the year from [31].

Since the installation of the system in Aysen, battery voltage measurements have been transmitted by the device to evaluate the reliability of PV panels as an energy source (Figure 6). Following installation, the battery remained fully charged, irrespective of frequent cloud coverage. Figure 7 provides evidence of the increasing cloud coverage during winter. Despite the reduced irradiance levels and temperatures, given the proximity of winter in the Southern Hemisphere, the developed system maintained enough energy to avoid entering the more stringent energy regimes, which implies a delay in file transmission. Hence, these results demonstrate the feasibility of year-round system operation without relying on conventional land connections, such as slip rings.

Figure 7 illustrates the cloud cover categories in Coyhaique, Aysen over the course of the year. Alongside the daily sunlight hours, the graph served as a basis for estimating the expected battery voltage in the upcoming months. Notably, the measurements recorded (Figure 6) followed the levels of cloud coverage (Figure 7) and daily sunlight patterns for the recorded days.

### 4.3. File Reception Assessment

The system has been operational for 187 days and has consistently delivered a file every 20 min. Initially, it was anticipated that the system would transmit a total of 13,451 files during this 187 day period. This estimation was performed considering that the system takes 20 min to capture and send measurements.

By analyzing the file reception depicted in Table 1, it is demonstrated that the number of files with a substantial delay (longer than the measurement window) was less than 0.3%. Additionally, two files were delivered ahead of schedule, likely due to the system’s connection and disconnection from the batteries during installation. By comparing the total number of received files with the estimation considering the uptime, it can be concluded that the system successfully transmitted 99.6% of the expected files, demonstrating how the system provides reliable data transmission.

### 4.4. Vibration Analysis

The initial vibration test involved conducting measurements on a single WT blade to extract its natural frequencies using the newly developed system. To determine the blade’s natural frequencies, damping ratio, and mode shape, operational modal analysis was performed on the recorded vibration data utilizing the deterministic-stochastic subspace identification method [32]. Following this analysis, clustering techniques were applied to identify and group correlated modes.

**Figure 8:**
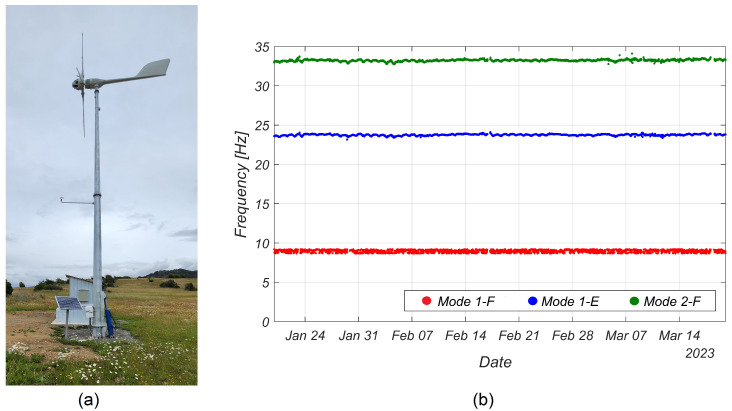
Low-capacity wind turbine installed in south Chile (WT1). (**a**) Image of the installed wind turbine. (**b**) Identified natural frequencies of three modes from mid-January to mid-March 2023.

Figure 8b illustrates three natural frequencies identified from the vibration data recorded on one of the blades of WT1 (see Figure 8a) using the deterministic-stochastic subspace identification method. In Figure 8b, 1-F and 2-F denote the first and second flapwise modes, respectively, while 1-E corresponds to the first edgewise mode. Subsequently, a comparison was made between these frequencies and the results obtained in [23]. Significantly, the extracted frequencies aligned closely with the previously reported results (which were obtained in a cantilever blade tested in a laboratory setting), providing compelling evidence of the system’s high accuracy in capturing the dynamic behavior of WT blades while using a reduced number of sensors. It is noted that although no damage was induced in WT1, changes in the identified modal frequencies can be observed in Figure 8b, which were due to environmental and operational variability. A detailed investigation of these effects is beyond the scope of this paper.

**Figure 9:**
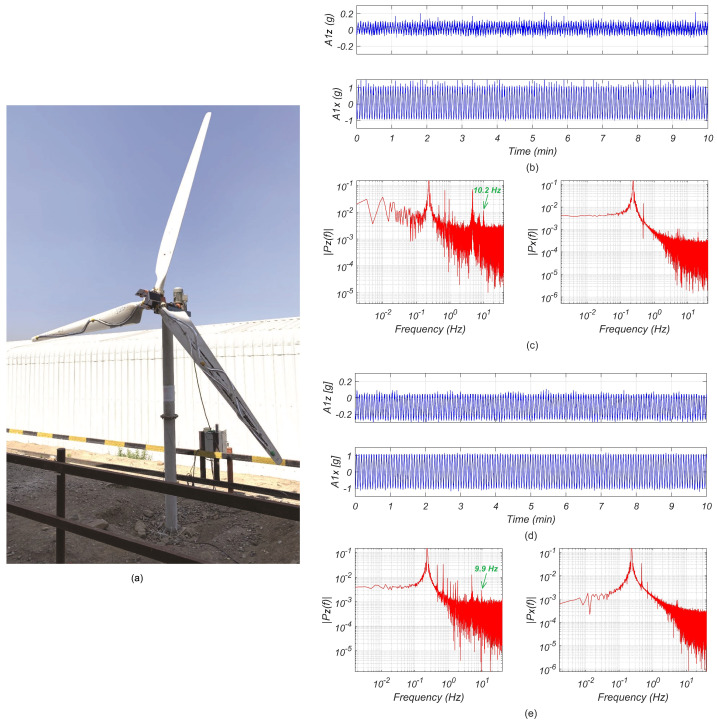
Vibration measurement sets for blades running at 15 r/min. (**a**) Image of the wind turbine simulation apparatus. (**b**) Healthy blade’s vibration signals from the *z* axis (**top**) and *x* axis (**bottom**). (**c**) FFTs for the *z* axis signal (**left**) and for the *x* axis signal (**right**) for the blade with no damage. (**d**) Damaged blade’s vibration signals from the *z* axis (**top**) and *x* axis (**bottom**). (**e**) FFTs for the *z* axis signal (**left**) and for the *x* axis signal (**right**) for the damaged blade.

Then, additional data necessary for referencing the extracted modal information from the installed turbines were obtained through baseline measurements using a wind turbine replica, which is displayed in Figure 9a. This apparatus was constructed using identical blades to those installed in the WT1 and WT2 systems. An electronic speed controller was incorporated into the replica axis for precise control. By measuring the vibrations on the blades under different operating conditions, such as varying speeds and pitch angles, additional masses, and damaged blades, a comprehensive control dataset was established for comparative analysis.

Figure 9b,c illustrates a set of measurements conducted on the blade of the wind turbine replica at a rotational speed of 15 r/min without any damage compared with the same configuration but with induced damage (Figure 9d,e). It is noted that the damage induced in one of the blades of the replica consisted of artificial cuts of varying severity at two specific locations along the blade. The recorded measurements on the *z* axis (i.e., flapwise) exhibited a clear difference in the vibration modes exhibited in the time series, whereas the damaged blade exhibited higher accelerations along the *z* axis, indicating a potential structural issue. This observation was further supported by conducting fast Fourier transform (FFT) analysis on the data. By comparing the FTTs of the undamaged and damaged cases, it can be observed that the peak corresponding to the frequency of the first flapwise mode (*z* axis) shifted from 10.2 Hz to 9.9 Hz, which was due to the loss of effective stiffness induced by the artificial cut in the blade.

**Figure 10:**
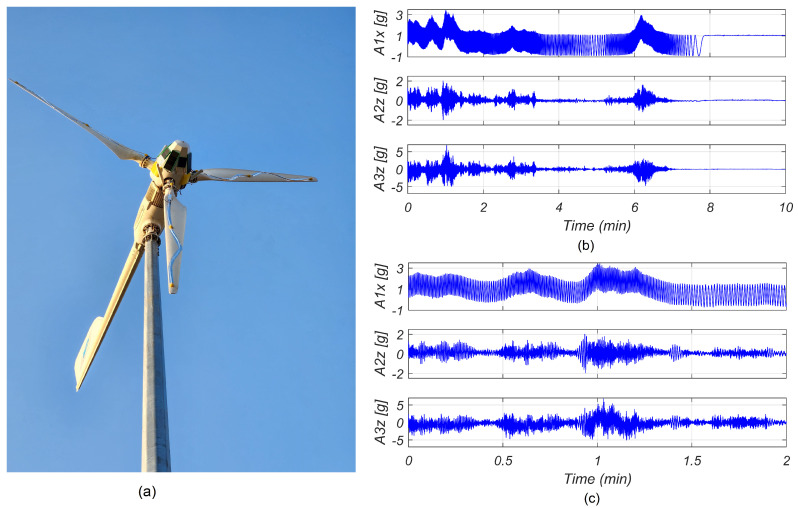
Acceleration measurements recorded on blades of WT2. (**a**) Image of the instrumented WT2. (**b**) The *x* axis acceleration of sensor A1 and *z* axis acceleration of sensors A2 and A3. (**c**) Zoomed-in version of signals in (**b**).

Figure 10 depicts the vibrations measured in WT2 on a particularly windy day. Notably, the turbine achieved high rotational speeds, exceeding 170 r/min. Of particular interest is the observation made by sensor A1, located at the root of the blade, along the x axis. A slight change in the offset can be observed as the speeds increased beyond a certain threshold. Due to the implementation of blade pitch speed control in the 5 kW turbines, when the speed surpassed the mentioned threshold, the blade adjusted its angle to modify the blade’s aerodynamic properties and decelerate the rotation. This change in angle results in an offset in the readings of the aforementioned sensor. The abrupt angle change is also evident on the z axis, where it abruptly reversed during the deceleration phase.

The data extracted from the experiments presented above provide sufficient information to develop a predictive model capable of detecting wind turbine blade damage.

## 5. Conclusions

In this article, we presented the design and implementation of a WT on-blade sensor system for remote vibration monitoring with a focus on structural damage identification. Through this work, we created and deployed an autonomous sensor system on two operational WTs, one of which is located in the far south of Chile and operates under harsh weather conditions throughout the year. We recorded baseline vibrations from this deployment to compare them with sample vibrations measured in an experimental three bladed system with induced damage. It was possible to observe a change in the frequencies captured by the device installed in the experimental set-up while demonstrating reliable data transmission from remote WTs, proving the ability to create a fully autonomous system capable of monitoring and evaluating the integrity of WTs for extended periods of time without human intervention, all while not compromising the low cost objective. Further research and development will be necessary in the future to create a complementary model capable of analyzing the data received from the system to detect and prevent structural damage. The data gathered in this study can serve as valuable training data for such models, which could then be combined with the presented device to create a damage detection system that ensures the safe and continuous operation of WTs.

## Figures and Tables

**Table 1:** File reception summary.

Month	Logged Minutes	Files Delayed	Anomalies	Files Expected	Files Received
November 2022	15,450	4	3	1549	1545
December 2022	22,250	4	7	2232	2225
January 2023	22,090	8	8	2232	2219
February 2023	20,060	6	7	2016	2006
March 2023	22,250	5	6	2232	2225
April 2023	21,350	8	7	2160	2151
May 2023	10,270	2	1	1030	1027
Total	133,980	37	39	13,451	13,398

## Data Availability

The data presented in this study are available on request from the corresponding authors.
